# The association between insulin levels and cortical bone: Findings from a cross-sectional analysis of pQCT parameters in adolescents

**DOI:** 10.1002/jbmr.1467

**Published:** 2011-11-16

**Authors:** Adrian Sayers, Debbie A Lawlor, Naveed Sattar, Jon H Tobias

**Affiliations:** 1 Musculoskeletal Research Unit, School of Clinical Sciences, University of BristolBristol, United Kingdom; 2 MRC Centre for Causal Analyses in Translational Epidemiology, School of Social and Community Medicine, University of BristolBristol, United Kingdom; 3 BHF Glasgow Cardiovascular Research Centre, Faculty of Medicine, University of GlasgowGlasgow, Scotland

**Keywords:** PERIOSTEAL CIRCUMFERENCE, BODY COMPOSITION, MUSCLE DENSITY, ALSPAC, INSULIN

## Abstract

Recent studies suggest that patients with type 2 diabetes mellitus are at increased risk of fracture, possibly because hyperinsulinemia is a risk factor for low bone mineral density, which may in turn be a consequence of a lipotoxic effect of visceral and/or intramuscular fat on bone. In the current study, we investigated whether insulin plays a role in cortical bone development by performing a cross-sectional study based on the Avon Longitudinal Study of Parents and Children (ALSPAC), where we examined associations between fasting insulin levels and peripheral quantitative computed tomography (pQCT) parameters as assessed at the mid-tibia in 2784 boys and girls with a mean age 15.5 years. In particular, we wished to examine whether associations that we observed were independent of body composition, including intramuscular fat. We found that insulin was inversely related to cortical bone mineral density (BMD_C_) after adjustment for age and after further adjustment for height, muscle cross-sectional area (MCSA), subcutaneous fat (SAT), and muscle density (MD), which is inversely related to intramuscular fat (−0.018, 95% confidence interval [CI] −0.030, −0.006, *p* < 0.0001). Insulin was positively related to periosteal circumference (PC) after adjusting for age (0.015, 95% CI 0.003, 0.027, *p* = 0.015; beta = change per 50% increase in insulin), but this changed to an inverse association after additional adjustment for height and body composition (−0.013, 95% CI −0.022, −0.003, *p* = 0.008). Path analyses revealed inverse associations between insulin and PC via a direct pathway (−0.012, 95% CI −0.022, −0.003, *p* = 0.01) and via MD (−0.002, 95% CI −0.004, −0.001, *p* = 0.0004), and positive associations between insulin and PC via SAT (0.013, 95% CI 0.009, 0.016, *p* < 0.0001) and MCSA (0.015, 95% CI 0.010, 0.020, *p* < 0.0001). In conclusion, we found an inverse relationship between insulin and PC via intramuscular fat, suggesting a lipotoxic effect on bone. However, an inverse association between insulin and both PC and BMD_C_ persisted after adjusting for all body composition variables, suggesting insulin also acts to inhibit bone development via additional pathways yet to be elucidated. © 2012 American Society for Bone and Mineral Research

## Introduction

Insulin resistance, the prevalence of which is increasing in adolescents alongside childhood obesity,^1^ is associated with several long-term complications of type 2 diabetes mellitus (DM) including an increased risk of fractures, particularly at the hip.^2^ Although bone mineral density (BMD) as measured by dual-energy X-ray absorptiometry (DXA) is inversely related to fracture risk in the general population,^3^ this relationship appears to be altered in type 2 DM, exemplified by findings from the Rotterdam study in which subjects with type 2 DM had an increased risk of nonvertebral fractures despite having a higher lumbar spine and femoral neck BMD.^4^ Consistent with the latter observation, several cross-sectional studies have found that BMD is also increased in patients with metabolic syndrome, which is associated with obesity, insulin resistance, and hyperinsulinemia.^5^–^9^ However, after adjustment for body mass index (BMI), BMD has frequently been found to be reduced in those with metabolic syndrome, particularly in males,^5^, ^7^–^9^ suggesting that, if anything, insulin may exert a negative influence on BMD once associations with BMI are taken into account.

Lipotoxicity describes the deposition of fat within the liver and skeletal muscles in association with obesity, which has been implicated in the pathogenesis of insulin resistance and other components of the metabolic syndrome.^10^ Any tendency for lipotoxicity to adversely affect bone might explain the negative association between metabolic syndrome and BMD in the studies described above, on the assumption that adjustment for BMI does not fully account for possible influences of visceral or intramuscular fat deposition. Consistent with this suggestion, visceral fat deposition, as assessed by computed tomography (CT) or magnetic resonance imaging (MRI), has been suggested to exert an adverse influence on BMD.^11^–^14^ The latter studies included two reports based on adolescent populations, suggesting that insulin also has the potential to impair childhood skeletal development via a lipotoxic pathway. Consistent with this suggestion, in the study of adolescents by Pollock and colleagues,^11^ HOMA-IR, a measure of insulin resistance, was also found to be inversely related to bone mineral content (BMC). Moreover, an inhibitory influence of visceral fat on bone was suggested to be on the same pathway as insulin because the negative association between visceral adipose tissue and BMC was no longer observed when HOMA-IR was included in the model.

One of the limitations in dissecting out relationships between insulin resistance, BMC, and fat deposition is the difficulty in adjusting for body size and stature, particularly in children where differences are exaggerated by variation in age and maturational status.^15^ Peripheral quantitative computed tomography (pQCT) overcomes this limitation by enabling effects on bone size to be distinguished from those on other parameters such as cortical bone density and the amount of trabecular bone. We recently used this method in the Avon Longitudinal Study of Parents and Children (ALSPAC) to explore relationships between body composition and bone geometry as measured by DXA and pQCT, respectively, observing that total body fat and lean mass both make strong independent contributions to cortical bone development.^16^ pQCT has also been used to evaluate possible adverse effects of lipotoxicity on skeletal development, based on measurement of intramuscular fat. For example, in a recent study of 444 girls aged 9 to 12 years, muscle density as measured by pQCT was positively related with cortical bone mineral density (BMD_C_), consistent with a negative association with the extent of intramuscular fat.^17^

In the current study, we explored the role of insulin in skeletal development in childhood by using pQCT to examine cross-sectional relationships between fasting insulin levels and indices of cortical bone development in adolescents from ALSPAC, in whom both these measures had been obtained at age 15 years. In particular, we wished to determine: 1) whether fasting insulin is related to cortical bone parameters at this age, 2) whether any relationships that we find are independent not only of lean mass and fat mass but also of intramuscular fat as reflected by muscle density, and 3) whether these associations show any evidence of gender specificity, in light of previous observations that after adjusting for BMI, BMD is preferentially reduced in male subjects with the metabolic syndrome.

## Materials and Methods

### Study population

ALSPAC is a geographically based birth cohort study investigating factors influencing the health, growth, and development of children. All pregnant women residing within a defined part of the former county of Avon in South West England with an expected date of delivery between April 1991 and December 1992 were eligible for recruitment, of whom 14,541 were enrolled (http://www.alspac.bristol.ac.uk).^18^ Ethical approval was obtained from the ALSPAC Law and Ethics committee and relevant local ethics committees. Data in ALSPAC is collected by self-completion postal questionnaires sent to parents, by linkage to computerized records, by abstraction from medical records, and from examination of the children at research clinics.

### Bone and anthropometric variables

BMC_C_ and cortical bone mineral density (BMD_C_) of the mid (50% from the distal endplate) right tibia were obtained using a Stratec XCT2000L (Stratec, Pforzheim, Germany) during the age 15.5-year research clinic to which all ALSPAC participants were invited as part of a study investigating the effects of physical activity on cortical bone as previously published.^19^ Periosteal circumference (PC) and endosteal circumference (EC) were derived using a circular ring model. Strength strain index (SSI) was calculated according to the Stratec user's manual.^20^ Cortical bone was defined using a threshold above 650 mg/cm^3^. Muscle cross-sectional area (MCSA), muscle density, and subcutaneous fat area (SAT) were obtained after image processing with a set of four convoluted image filters, as described in the Stratec user's manual.^20^ In brief, image convolution processes the original image I and creates I'; if a second filter is applied, I' is processed and creates I” and so on. Using the Stratec large soft tissue filters (F03F05F05 = 3 × 3 [−500, 500 mg/cm^3^], 5 × 5 [−500, 300 mg/cm^3^]), the image was sequentially smoothed three times and total limb cross-sectional area (CSA) was calculated. A subsequent filter was applied to remove all fat density voxels from the image (F07 = [−500, 30 mg/cm^3^]), and total fat-free limb CSA was calculated. MCSA was calculated by subtracting total bone area from the fat-free total limb CSA. Within Subject coefficient of variation for pQCT parameters are displayed in parentheses: tibial length (4.04%), BMC_C_ (2.71%), BMD_C_ (1.29%), PC (1.58%), EC (4.03%), MCSA (6.73%), MDEN (5.24%), SAT (4.86%), SSI (3.72%). Height was measured using a Harpenden stadiometer (Holtain Ltd., Crymych, UK). All densitometric and anthropometric measures were taken in the 15.5-year research clinic.

### Confounding variables

Puberty was assessed using a self-completed Tanner stage questionnaire (pubic hair domain) at a mean age of 14.8 years.^21^ Social economic position was derived using the registrar general social class classification and maternal highest educational attainment.^22^

### Insulin measurement

Participants were asked to fast overnight (for those attending in the morning) or for a minimum of 6 hours for those attending after lunch. Blood plasma samples (EDTA) were immediately spun and frozen at −80°C. Measurements were assayed shortly (3 to 9 months) after samples were taken with no previous freeze-thaw cycles. Insulin was measured by an ultrasensitive ELISA (Mercodia, Uppsala, Sweden) automated microparticle enzyme immunoassay that does not cross-react with proinsulin. Its sensitivity was 0.07 mU/L, and inter- and intra-assay coefficients of variation were <6%.

### Statistical analysis

Descriptive statistics are presented as means, standard deviations, and interquartile cut-points. Insulin, muscle density, and SAT were positively skewed and subsequently log_e_ transformed to aid model specification. Ordinary least squares (OLS) linear regression was used to investigate associations between insulin and pQCT variables after minimal adjustment (age only) and adjustment for height, MCSA, muscle density, and SAT. Because of the strong association between EC and PC, analyses with EC were adjusted for PC. Models with BMD_C_ were analyzed with and without adjustment for EC and PC because of relationships between BMD_C_ and cortical bone size. Path analyses were conducted to explore the relationship between insulin and PC. The direct association was obtained from a multivariate linear regression of exposure (ie, insulin) on outcome (ie, PC) adjusted for confounders. Indirect associations (via MCSA, muscle density, and SAT) were calculated by multiplying the association of exposure on intermediate adjusted for confounders, with the association of the intermediate on outcome adjusted for confounders. The total effect is the sum of the direct and indirect associations.^23^ Path coefficients were calculated using a nonparametric bootstrap procedure, stratified by sex and sampled with replacement for 10,000 iterations. Sensitivity analyses were conducted, adjusted for pubertal status, maternal education, and maternal and paternal social class. Data are assumed to be missing at random, and complete case analysis was performed in all cases. All analyses were conducted in STATA 11.1 MP (StataCorp, College Station, TX, USA).

## Results

**Fig. 1:**
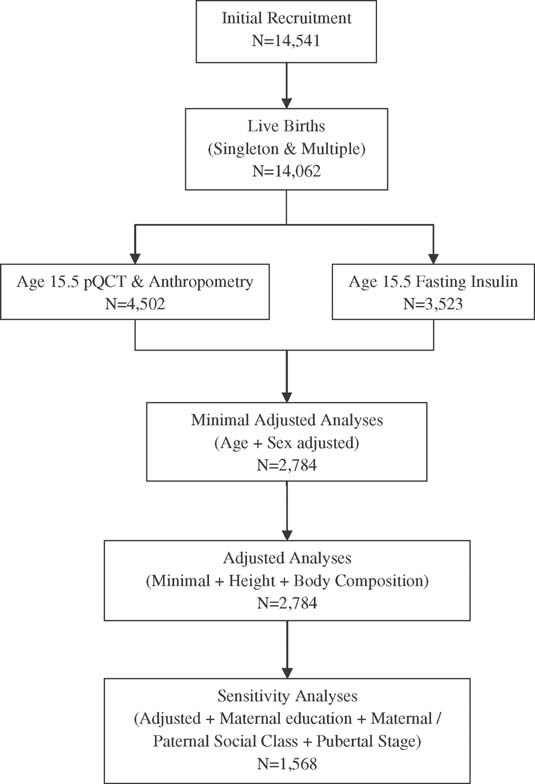
Diagram showing the source of subjects for main analyses and for sensitivity analyses based on the subset of subjects for which information was available on Tanner stage and socioeconomic status.

The study included 1344 boys and 1440 girls (see Fig. 1 for recruitment flow). As shown in Table 1, fasting insulin levels were higher in girls. As expected, boys had greater lean mass as reflected by greater MSCA, whereas fat mass as reflected by SAT was greater in girls. In contrast, muscle density was similar in boys and girls. Boys were taller and had greater inner and outer circumferences of tibial cortical bone, as reflected by EC and PC, respectively. BMD_C_ was greater in girls, whereas BMC_C_ was greater in boys, reflecting the fact that variation in this parameter is largely determined by differences in cortical bone size. A total of 729 boys and 839 girls were available with information on socioeconomic variables and pubertal status (Supplemental Table S1), which revealed that 84% of boys and 90% of girls were in advanced puberty as defined by Tanner stage IV/V.

**Table 1:** Characteristics of the 1344 Boys and 1440 Girls Included in the Study^a^k

		Mean	(SD)	25th centile	50th centile	75th centile
Age (years)	Boys	15.47	(0.3)	15.3	15.4	15.6
	Girls	15.48	(0.3)	15.3	15.4	15.6
Insulin (IU/mL)	Boys	9.07	(5.3)	6.1	8.1	10.9
	Girls	10.62	(5.4)	7.4	9.7	12.8
Height (cm)	Boys	174.73	(7.6)	170	175	179.8
	Girls	164.77	(6.1)	161	164.6	168.7
Weight (kg)	Boys	64.05	(11.3)	56.8	62.6	69.8
	Girls	59.35	(10.4)	52.3	57.7	64.5
BMI (kg/m^2^)	Boys	20.91	(3.0)	18.9	20.3	22.2
	Girls	21.84	(3.5)	19.4	21.2	23.4
MCSA (mm^2^)	Boys	5538.45	(1051.8)	4809.8	5459.3	6192.5
	Girls	5001.25	(893.9)	4386.4	4938.6	5575.5
SAT (mm^2^)	Boys	2053.33	(726.2)	1567	1906.4	2378.4
	Girls	2913.39	(826.1)	2332.8	2789.5	3356
MD (mg/cm^3^)	Boys	85.36	(4.7)	82.4	84.4	87.3
	Girls	85.17	(5.0)	81.8	84	87.1
PC (mm)	Boys	76.42	(5.3)	72.9	76.3	79.7
	Girls	69.55	(4.8)	66.2	69.2	72.6
EC (mm)	Boys	40.9	(5.6)	37.3	40.6	44
	Girls	36.88	(5.1)	33.5	36.3	39.6
BMD_C_ (mg/cm^3^)	Boys	1077.13	(33.2)	1056.7	1078.9	1101.5
	Girls	1124.61	(22.6)	1110.9	1125.9	1139.8
BMC_C_ (mg/mm)	Boys	357.1	(52.1)	321.8	356.1	388.9
	Girls	310.86	(41.0)	282.5	310.1	338.2
SSI (mm^3^)	Boys	1172.24	(231.6)	1011.1	1159.2	1324
	Girls	925.39	(179.5)	798.5	904.7	1040.3

SD = standard deviation; BMI = body mass index; MCSA = muscle cross-sectional area; SAT = subcutaneous fat area; MD = muscle density; PC = periosteal circumference; EC = endosteal circumference; BMD_C_ = cortical bone density; BMC_C_ = cortical bone mineral content; SSI = strength strain index.

a Peripheral quantitative computed tomography (pQCT)-derived variables obtained at the mid-tibia.

The relationship between fasting insulin and indices of body composition as assessed by pQCT were explored in analyses adjusted for age and height (Table 2). Insulin was positively related to both MSCA and SAT. The relationship between insulin and SAT was stronger in boys (*p* < 0.05 for sex interaction). Insulin was inversely related to muscle density, in keeping with a positive association with intramuscular fat, with evidence that this association was also stronger in boys (*p* = 0.09 for sex interaction; muscle density was also adjusted for MCSA in view of the positive relationship between these two parameters, independent of height). Equivalent results were obtained when results were analyzed in the subset of subjects with available information on socioeconomic status and puberty followed by adjustment for these variables (Supplemental Table S2).

**Table 2:** Associations Between Fasting Insulin and Body Composition Variables Derived From Tibial pQCT in 1344 Boys and 1440 Girls

Outcome	Adjusted^a^	Sex	Beta^b^	(95% CI)	*p* Value	*p* Value_(Sex Dif.)_
Height	Age	Boys	0.010	(−0.006, 0.027)	0.2276	0.5397
		Girls	0.002	(−0.015, 0.021)	0.7805	
		All	0.007	(−0.005, 0.019)	0.2601	
MCSA	Height + Age	Boys	0.033	(0.014, 0.052)	0.0007	0.2405
		Girls	0.050	(0.029, 0.071)	0.0001	
		All	0.041	(0.027, 0.055)	0.0001	
SAT	Height + Age	Boys	0.094	(0.078, 0.110)	0.0001	0.0505
		Girls	0.070	(0.052, 0.088)	0.0001	
		All	0.083	(0.071, 0.095)	0.0001	
MD	MCSA + Height + Age	Boys	−0.023	(−0.034, −0.013)	0.0001	0.0929
		Girls	−0.010	(−0.021, 0.001)	0.0844	
		All	−0.017	(−0.025, −0.010)	0.0001	

pQCT = peripheral quantitative computed tomography; CI = confidence interval; MCSA = muscle cross-sectional area; SAT = subcutaneous fat area; MD = muscle density.

a Covariates used for each model.

b Beta coefficients represent standard deviation change in the outcome variable per 50% increase in fasting insulin.

Relationships between insulin and pQCT parameters were subsequently examined in minimally adjusted models (age) and more completely adjusted models (age, height, MSCA, SAT, and muscle density) (Table 3). In minimally adjusted analyses, insulin was positively related to PC. There was some evidence that this relationship was stronger in girls, in whom beta coefficients were more than twice that in boys, although the *p* value for sex interaction was >0.1. Conversely, in our more completely adjusted model, insulin was inversely related to PC, with a suggestion that this relationship was stronger in boys based on beta coefficients, although the *p* value for sex interaction was again >0.1. Insulin was unrelated to EC in either minimal or more completely adjusted analyses (EC was adjusted for PC in all models, so as to provide an estimate of cortical thickness). Insulin was inversely related with BMD_C_, in both minimal and more completely adjusted analyses. These included further analyses in which associations were adjusted not only for age and body composition but also for PC and EC, in view of associations between the latter parameters and BMD_C_.^16^ There was also evidence that the inverse association between insulin and BMD_C_ was stronger in boys, in whom beta coefficients were approximately twice that in girls, although the *p* value for sex interaction was ≥0.2. Similar results for BMC_C_ and SSI were observed to those for PC. Equivalent results were obtained when results were analyzed in the subset of subjects with available information on socioeconomic status and puberty followed by adjustment for these variables (Supplemental Table S3).

**3:** Associations Between Insulin and 50% Mid-Tibia Cortical Bone Variables^a^

Outcome	Adjusted^b^	Sex	Beta^c^	(95% CI)	*p* Value	*p V*alue _(Sex Dif.)_
PC	Age	Boys	0.009	(−0.008, 0.025)	0.3029	0.2687
		Girls	0.023	(0.004, 0.041)	0.0141	
		All	0.015	(0.003, 0.027)	0.0152	
	Age, Height, MCSA, SAT, MD	Boys	−0.017	(−0.029, −0.004)	0.0119	0.3911
		Girls	−0.008	(−0.022, 0.006)	0.2390	
		All	−0.013	(−0.022, −0.003)	0.0077	
EC	PC, Age	Boys	0.000	(−0.013, 0.014)	0.9897	0.3776
		Girls	−0.009	(−0.024, 0.006)	0.2351	
		All	−0.004	(−0.014, 0.006)	0.4463	
	PC, Age, Height, MCSA, SAT, MD	Boys	0.001	(−0.013, 0.014)	0.9190	0.6003
		Girls	−0.004	(−0.019, 0.010)	0.5341	
		All	−0.001	(−0.011, 0.009)	0.7953	
BMD_C_	Age	Boys	−0.025	(−0.041, −0.009)	0.0023	0.2673
		Girls	−0.011	(−0.028, 0.006)	0.1933	
		All	−0.018	(−0.030, −0.006)	0.0023	
	Age, Height, MCSA, SAT, MD	Boys	−0.024	(−0.040, −0.008)	0.0035	0.2769
		Girls	−0.011	(−0.028, 0.006)	0.2138	
		All	−0.018	(−0.030, −0.006)	0.0026	
BMD_C_	PC, EC, Age	Boys	−0.024	(−0.039, −0.009)	0.0018	0.2073
		Girls	−0.010	(−0.026, 0.006)	0.2459	
		All	−0.017	(−0.028, −0.006)	0.0020	
	PC, EC, Age, Height, MCSA, SAT, MD	Boys	−0.030	(−0.045, −0.014)	0.0001	0.1997
		Girls	−0.015	(−0.031, 0.001)	0.0687	
		All	−0.023	(−0.034, −0.012)	0.0001	
BMC_C_	Age	Boys	0.003	(−0.015, 0.021)	0.7286	0.1757
		Girls	0.021	(0.002, 0.041)	0.0306	
		All	0.012	(−0.002, 0.025)	0.0821	
	Age, Height, MCSA, SAT, MD	Boys	−0.018	(−0.032, −0.003)	0.0156	0.2575
		Girls	−0.006	(−0.021, 0.010)	0.4772	
		All	−0.013	(−0.024, −0.002)	0.0197	
SSI	Age	Boys	0.006	(−0.011, 0.024)	0.4695	0.2238
		Girls	0.022	(0.003, 0.041)	0.0203	
		All	0.014	(0.001, 0.026)	0.0331	
	Age, Height, MCSA, SAT, MD	Boys	−0.020	(−0.033, −0.007)	0.0021	0.1829
		Girls	−0.007	(−0.021, 0.006)	0.2932	
		All	−0.015	(−0.024, −0.005)	0.0026	

MSCA = muscle cross sectional area; SAT = subcutaneous fat area; MD = muscle density; PC = periosteal circumference; EC = endosteal circumference; BMD_C_ = cortical bone density; BMC_C_ = cortical bone mineral content; SSI= strength strain index.

a Associations between fasting insulin and cortical bone parameters derived from tibial pQCT, in 1344 boys and 1440 girls.

b Covariates used for each model are listed under adjusted.

c Beta coefficients represent SD change in the outcome variable per 50% increase in fasting insulin.

**Fig. 2:**
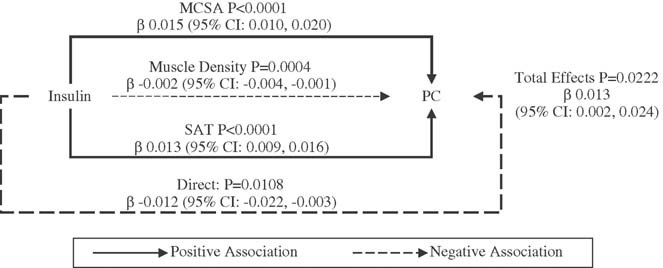
Path diagram depicting relationships between fasting insulin and periosteal circumference (PC) in 1344 boys and 1440 girls combined. The model is constrained to show the effects via muscle cross-sectional area (MSCA), muscle density, subcutaneous fat area (SAT), and a “direct” pathway after adjusting for body composition. Solid lines depict positive effects and broken lines depict negative effects. The thickness of the line represents the strength of each pathway, reflected by beta (β) estimates, which were derived from regressions between fasting insulin and indices of body composition as shown in Table 2 and between indices of body composition and PC as follows: MCSA versus PC, beta = 0.175 (95% CI 0.161 to 0.189); SAT versus PC, beta = 0.043 (95% CI 0.031 to 0.054); muscle density versus PC, beta = 0.025 (95% CI 0.007 to 0.043).

**Fig. 3:**
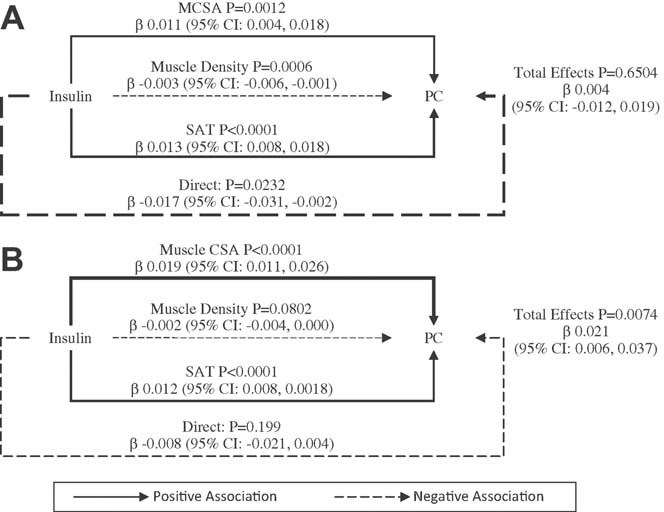
Path diagram depicting relationships between fasting insulin and periosteal circumference (PC) in (*A*) 1344 boys and (*B*) 1440 girls. The model is constrained to show the effects via muscle cross-sectional area (MSCA), muscle density, subcutaneous fat area (SAT), and a “direct” pathway after adjusting for body composition. Solid lines depict positive effects and broken lines depict negative effects. The thickness of the line represents the strength of each pathway, reflected by beta (β) estimates, which were derived from regressions between fasting insulin and indices of body composition as shown in Table 2 and between indices of body composition and PC. Boys: MCSA versus PC, beta = 0.161 (95% CI 0.142 to 0.18); SAT versus PC, beta = 0.034 (95% CI 0.019 to 0.049); muscle density versus PC, beta = 0.02 (95% CI −0.004 to 0.044). Girls: MCSA versus PC, beta = 0.191 (95% CI 0.17 to 0.212); SAT versus PC, beta = 0.053 (95% CI 0.035 to 0.071); muscle density versus PC, beta = 0.03 (95% CI 0.002 to 0.057).

The change in direction of association between insulin and PC/BMC_C_ according to method of adjustment suggests this relationship involves a combination of direct effects and indirect effects secondary to changes in body composition. To explore these, path analyses were performed, comparing the overall effect of insulin on PC via a direct pathway independent of body composition and via SAT, muscle density, and MCSA. In analyses based on boys and girls combined, there was an inverse association between insulin and PC via a direct pathway and an inverse association between insulin and PC via muscle density (Fig. 2). In contrast, there were positive associations between insulin and PC involving SAT and MCSA pathways. In further path analyses based on boys and girls separately, the inverse association between insulin and PC via a direct pathway appeared to be stronger in boys, whereas the positive association between insulin and PC via MCSA tended to be stronger in girls (Fig. 3). Consequently, whereas there was a net positive association between insulin and PC in girls, no net effect was seen in boys.

## Discussion

We performed a cross-sectional analysis of the relationship between insulin levels and pQCT parameters as assessed at the mid-tibia in 2784 boys and girls mean 15.5 years of age. We found that insulin was positively related to tibial BMC_C_ in minimally adjusted models, which is likely to reflect an influence on periosteal growth because an equivalent positive association was observed with tibial PC. However, the positive relationship between insulin levels and PC in minimally adjusted analyses appears to be confounded by associations with fat and lean mass because an inverse association was observed after adjusting for these. There was also evidence for an inverse pathway between insulin and PC via intramuscular fat as reflected by muscle density, but pathway analyses suggested that this was relatively weak, and an inverse association between insulin and PC was still observed after adjustment for all body composition variables including muscle density. In addition, insulin levels were inversely related to BMD_C_ in both minimally and more completely adjusted analyses, consistent with our previous observation that unlike PC, BMD_C_ only shows very weak relationships with fat mass and lean mass.^16^

Presumably, the suggestion of positive confounding of the relationship between insulin and PC by body composition is explained by associations between body composition and both insulin and PC. For example, we observed a positive association between insulin and tibial MSCA and SAT, in line with previous findings that insulin levels are positively related to indices of body composition including in adolescents.^24^ Moreover, we recently reported that equivalent estimates of these body composition parameters assessed by total body DXA have strong positive relationships with tibial PC.^16^ Subsequent path analyses indicated that broadly effects via muscle and fat contribute equally to this indirect positive effect of insulin on PC via body composition. Conversely, any evidence that negative confounding by intramuscular fat as reflected by muscle density contributed to the relationship between insulin and PC is explained by an inverse relationship between insulin and muscle density, combined with a positive association between muscle density and PC (Figs. 2 and 3).

Because PC and BMD_C_ both represent positive determinants of bone mass as measured by DXA, our findings are consistent with previous reports that after adjustment for BMI, BMD and/or BMC as measured by DXA is reduced in subjects with elevated insulin levels secondary to insulin resistance in the context of the metabolic syndrome, including adolescents and prepubertal children.^5^–^9^, ^25^ In the majority of these studies, inverse associations between insulin and BMD after adjusting for BMI appeared to be stronger in males, which also applied to our findings of associations between insulin and both PC and BMC_C_. In addition, our results are consistent with previous pQCT-based studies in older adults with raised insulin levels in the context of type 2 DM, in whom cortical bone size was reduced after adjusting for BMI/weight,^26^, ^27^ consistent with a reduction in PC. Interestingly, a recent high-resolution pQCT study in type 2 DM suggested that this condition is also associated with an increase in cortical porosity,^28^ suggesting that the inverse association we observed between insulin and BMD_C_ may be secondary to an increase in cortical bone turnover.

Taken with results of previous studies based in adult populations, our findings suggest that raised insulin levels are associated with a negative influence on cortical bone when associated changes in body composition are taken into account, which can manifest in childhood and may persist throughout the remainder of the life course. Therefore, it is tempting to speculate that the inverse association between insulin levels and cortical bone parameters that we observed after adjusting for fat mass, lean mass, and muscle density contribute to the increase in fracture risk described in older patients with type 2 DM.^2^ Interestingly, this increased risk appears to be greatest for fractures at sites such as the lower leg,^29^, ^30^ consistent with our results based on pQCT of the tibia. The suggestion that any adverse influence of insulin on fracture risk is largely independent of intramuscular fat is also consistent with findings from a study of older subjects with type 2 DM, in whom the observed increase in fracture risk was not attenuated by adjustment for intramuscular fat as assessed by CT.^31^

The current findings are based on analysis of fasting insulin, rather than an index of insulin resistance such as homeostasis model assessment of insulin resistance (HOMA-IR), which might have more relevance to type 2 DM in which insulin resistance is the cardinal feature. However, glucose levels are tightly controlled in childhood, and fasting insulin is highly correlated with HOMA-IR (as confirmed by a bivariate Pearson correlation of *r* = 0.98 between fasting insulin and HOMA-IR in the current study population), so HOMA-IR is not thought to offer any advantages in evaluating insulin resistance in children.^32^

In terms of the mechanisms underlying the apparent adverse effect of insulin on cortical bone suggested by our results, if anything, insulin and related hormones such as insulin-like growth factor 1 are thought to exert a trophic effect on the skeleton.^33^, ^34^ Therefore, any adverse influence of insulin on cortical bone growth is likely to reflect a role of other factors associated with raised insulin levels rather than a direct action of insulin itself. One such candidate is adiponectin, which is inversely related to insulin resistance, but we recently observed an inverse association between this factor and cortical bone area in ALSPAC, suggesting that if anything, this pathway contributes a positive rather than negative influence.^35^ Another potential pathway is between insulin and osteocalcin, in light of animal studies that suggest that osteoblast-derived uncarboxylated osteocalcin acts to improve insulin sensitivity,^36^, ^37^ which has been confirmed in studies in childhood in which uncarboxylated osteocalcin levels were found to be lower in those with prediabetes/insulin resistance.^38^ However, to the extent that a relationship exists between bone and energy metabolism, how reduced levels of uncarboxylated osteocalcin in the context of hyperinsulinemia might affect osteoblast function and cortical bone growth or turnover is currently unclear.

### Limitations

Given its cross-sectional design, it is difficult to infer causal relationships between the biological variables investigated. Moreover, our results are solely reliant on pQCT measurements obtained at a single site, namely the mid-tibia. In a previous study of relationships between obesity and pQCT outcomes, a positive relationship was observed between fat mass and cortical bone measures at the tibia but not the radius;^39^ if related factors such as insulin also vary in terms of relationships with cortical bone indices of the upper and lower limb, it may be that our results are only applicable to lower limb development. Furthermore, we were unable to examine relationships with trabecular bone, which represents a limitation in light of evidence that type 2 DM may be associated with increased trabecular BMD.^28^ However, in a further study in ALSPAC, we observed an equivalent inverse association between fasting insulin and total body BMD as measured by DXA after adjusting for fat and lean mass, suggesting that the relationship described here based on tibial pQCT is representative of associations between fasting insulin and bone development in the skeleton as a whole (our unpublished observations). Finally, we only had limited information on potentially important confounders such as puberty, which was assessed by self-completion questionnaire as opposed to more direct clinical evaluation.

### Final conclusions

In conclusion, we have found that, after accounting for associated differences in body composition, insulin levels are inversely related to both tibial PC and BMD_C_. Although insulin levels were positively associated with intramuscular fat deposition as reflected by muscle density, consistent with a possible lipotoxic pathway, the inverse relationship between insulin and cortical bone parameters persisted after adjusting for both subcutaneous and intramuscular fat. Further investigation is justified to clarify the mechanisms underlying this apparent negative influence of insulin on cortical bone development because any tendency for insulin to impair cortical bone development may have important clinical consequences, such as contributing to the excess in fracture risk observed in conditions associated with hyperinsulinemia such as type 2 DM.

## Disclosures

All authors state that they have no conflicts of interest.
