# Supplementary material for: The association between insulin levels and cortical bone: Findings from a cross-sectional analysis of pQCT parameters in adolescents
Source: J Bone Miner Res. 2011 Nov 16;27(3):610–8. doi: 10.1002/jbmr.1467 (PMC3378703; doi:10.1002/jbmr.1467)
Supplement: Supplementary file 1 [file jbmr0027-0610-SD1.doc]

**SUPPLEMENTARY TABLE 1: Puberty and socio-economic status**

| Variable | Category | Boys | Girls | All |
| --- | --- | --- | --- | --- |
| Maternal Education | A level | 237 | 261 | 498 |
|  | CSE | 41 | 77 | 118 |
|  | Degree | 175 | 188 | 363 |
|  | O level | 242 | 277 | 519 |
|  | Vocational | 34 | 36 | 70 |
| Social Class (Maternal) | i | 79 | 66 | 145 |
|  | ii | 292 | 336 | 628 |
|  | iii | 269 | 308 | 577 |
|  | iv | 41 | 57 | 98 |
|  | v | 48 | 72 | 120 |
| Social Class (Paternal) | i | 125 | 127 | 252 |
|  | ii | 290 | 314 | 604 |
|  | iii | 85 | 117 | 202 |
|  | iv | 177 | 206 | 383 |
|  | v | 52 | 75 | 127 |
| Tanner Stage (Pubic Hair) | i + ii + iii | 114 | 81 | 195 |
|  | iv | 392 | 415 | 807 |
|  | v | 223 | 343 | 566 |
| Total |  | 729 | 839 | 1568 |

Table shows breakdown according to Tanner stage and socio-economic status, in the subset of the study sample where this additional information was available.

**SUPPLEMENTARY TABLE 2: Associations between insulin and body composition (adjusted for puberty and socio-economic status)**

| Exposure | Adjusted | Sex | Beta | (95% CI) | | p-value | p-value(Sex. Dif.) |
| --- | --- | --- | --- | --- | --- | --- | --- |
| Height | Age SAT, MSCA, MD | Boys | 0.009 | (-0.013, | 0.030) | 0.4436 | 0.5885 |
|  |  | Girls | 0.000 | (-0.024, | 0.023) | 0.9786 |  |
|  |  | **All** | **0.003** | **(-0.013,** | **0.019)** | **0.7262** |  |
| MCSA | Age, SAT, MD, Height | Boys | 0.044 | (0.018, | 0.069) | 0.0008 | 0.8366 |
|  |  | Girls | 0.040 | (0.012, | 0.067) | 0.0047 |  |
|  |  | **All** | **0.041** | **(0.022,** | **0.060)** | **0.0001** |  |
| SAT | Age, MCSA, Height | Boys | 0.113 | (0.090, | 0.135) | 0.0001 | 0.0003 |
|  |  | Girls | 0.053 | (0.029, | 0.077) | 0.0001 |  |
|  |  | **All** | **0.086** | **(0.069,** | **0.102)** | **0.0001** |  |
| MDEN | Age, MCSA, Height | Boys | -0.022 | (-0.036, | -0.008) | 0.0022 | 0.0996 |
|  |  | Girls | -0.005 | (-0.020, | 0.011) | 0.5436 |  |
|  |  | **All** | **-0.014** | **(-0.024,** | **-0.004)** | **0.0086** |  |

Associations between fasting insulin, height and body composition variables derived from tibial pQCT, in 729 boys and 839 girls with information on Tanner stage and socio-economic status for which analyses were also adjusted. Beta coefficients represent SD change in the outcome variable per 50% increase in fasting insulin. Additional covariates used for each model are listed under ‘adjusted’. MSCA = muscle cross sectional area; SAT = subcutaneous fat area; MD = muscle density.

**SUPPLEMENTARY TABLE 3: Associations between insulin and bone variables (adjusted for puberty and socio-economic status)**

| Outcome | Adjusted | Sex | Beta | (95% CI) | | p-value | p-value _(Sex. Dif.)_ |
| --- | --- | --- | --- | --- | --- | --- | --- |
| PC | Age | Boys | 0.015 | (-0.007, | 0.038) | 0.1849 | 0.4295 |
|  |  | Girls | 0.002 | (-0.022, | 0.026) | 0.8781 |  |
|  |  | **All** | **0.008** | **(-0.008,** | **0.025)** | **0.3286** |  |
|  | Age, Height, MCSA, SAT, MD | Boys | -0.017 | (-0.035, | 0.001) | 0.0676 | 0.7121 |
|  |  | Girls | -0.022 | (-0.041, | -0.003) | 0.0228 |  |
|  |  | **All** | **-0.019** | **(-0.032,** | **-0.006)** | **0.0035** |  |
| EC | PC, Age | Boys | 0.002 | (-0.017, | 0.020) | 0.8488 | 0.3964 |
|  |  | Girls | -0.010 | (-0.030, | 0.010) | 0.3267 |  |
|  |  | **All** | **-0.003** | **(-0.016,** | **0.011)** | **0.6839** |  |
|  | PC, Age, Height, MCSA, SAT, MD | Boys | -0.002 | (-0.020, | 0.016) | 0.8148 | 0.7071 |
|  |  | Girls | -0.007 | (-0.026, | 0.012) | 0.4548 |  |
|  |  | **All** | **-0.003** | **(-0.016,** | **0.010)** | **0.6116** |  |
| BMD_C_ | Age | Boys | -0.023 | (-0.043, | -0.003) | 0.0263 | 0.4708 |
|  |  | Girls | -0.012 | (-0.034, | 0.010) | 0.2797 |  |
|  |  | **All** | **-0.019** | **(-0.034,** | **-0.004)** | **0.0102** |  |
|  | Age, Height, MCSA, SAT, MD | Boys | -0.018 | (-0.039, | 0.003) | 0.0994 | 0.6221 |
|  |  | Girls | -0.010 | (-0.032, | 0.012) | 0.3646 |  |
|  |  | **All** | **-0.015** | **(-0.031,** | **0.000)** | **0.0482** |  |
| BMD_C_ | PC, EC, Age | Boys | -0.019 | (-0.039, | 0.000) | 0.0462 | 0.7102 |
|  |  | Girls | -0.014 | (-0.034, | 0.006) | 0.1783 |  |
|  |  | **All** | **-0.019** | **(-0.033,** | **-0.005)** | **0.0091** |  |
|  | PC, EC, Age, Height, MCSA, SAT, MD | Boys | -0.024 | (-0.044, | -0.004) | 0.0175 | 0.7399 |
|  |  | Girls | -0.019 | (-0.040, | 0.001) | 0.0667 |  |
|  |  | **All** | **-0.023** | **(-0.037,** | **-0.009)** | **0.0018** |  |
| BMC_C_ | Age | Boys | 0.007 | (-0.017, | 0.030) | 0.5737 | 0.9316 |
|  |  | Girls | 0.005 | (-0.020, | 0.031) | 0.6852 |  |
|  |  | **All** | **0.004** | **(-0.013,** | **0.022)** | **0.6289** |  |
|  | Age, Height, MCSA, SAT, MD | Boys | -0.015 | (-0.034, | 0.005) | 0.1434 | 0.9284 |
|  |  | Girls | -0.013 | (-0.034, | 0.007) | 0.1977 |  |
|  |  | **All** | **-0.015** | **(-0.030,** | **-0.001)** | **0.0353** |  |
| SSI | Age | Boys | 0.012 | (-0.011, | 0.034) | 0.3101 | 0.5572 |
|  |  | Girls | 0.002 | (-0.023, | 0.026) | 0.8873 |  |
|  |  | **All** | **0.006** | **(-0.011,** | **0.022)** | **0.5085** |  |
|  | Age, Height, MCSA, SAT, MD | Boys | -0.020 | (-0.038, | -0.002) | 0.0259 | 0.9968 |
|  |  | Girls | -0.020 | (-0.039, | -0.002) | 0.0319 |  |
|  |  | **All** | **-0.020** | **(-0.033,** | **-0.008)** | **0.0018** |  |

Associations between fasting insulin and cortical bone parameters derived from tibial pQCT, in 729 boys and 839 girls with information on Tanner stage and socio-economic status for which analyses were also adjusted. Beta coefficients represent SD change in the outcome variable per 50% increase in fasting insulin. Additional covariates used for each model are listed under ‘adjusted’. MSCA = muscle cross sectional area; SAT = subcutaneous fat area; MD = muscle density; PC = periosteal circumference; EC = endosteal circumference; BMD_C_ = cortical bone density; BMC_C_ = cortical bone mineral content; SSI= strength strain index.
